# Biochemical properties of immobilized horseradish peroxidase on ceramic and its application in removal of azo dye

**DOI:** 10.1038/s41598-024-78998-9

**Published:** 2024-11-15

**Authors:** Hala A. Salah, Alshaimaa M. Elsayed, Azza M. Abdel-Aty, Gamal A. Khater, Amany A. El-Kheshen, Mohammad M. Farag, Saleh A. Mohamed

**Affiliations:** 1 Molecular Biology Department, National Research Centre, Dokki, Cairo, Egypt; 2 Glass Research Department, National Research Centre, Cairo, 12622 Egypt

**Keywords:** Horseradish peroxidase, Ceramics, Immobilization, Characterization, Decolorization, Biochemistry, Biotechnology

## Abstract

In the current work, electrostatic interactions were used to immobilize the horseradish peroxidase (HRP) onto five types of ceramic materials (C) with different concentrations of oxidized metals (C1–C5). The highest immobilization efficiency (70 and 77%) was detected at 6 mg C3 and 18 enzyme units. Scanning Electron Microscope (SEM), Energy Dispersive X-ray (EDX) and Fourier Transform Infrared (FTIR) analysis of C3-HRP confirmed the immobilization of the enzyme. After ten reuses, the reusability analysis showed that (66%) of the C3-HRP enzyme activity was retained. For C3-HRP, the optimum pH and temperature of the soluble enzyme were shifted from 7.0 and 30 °C to 6.0 and 50 °C. Up to 40 °C and 50 °C, respectively, the soluble HRP and C3-HRP remained steady. The kinetic analysis revealed that the Km and Vmax of soluble HRP and C3-HRP were, respectively, 5.5 mM, 0.66 units, and 8 mM, 0.52 units for hydrogen peroxide (H_2_O_2_) and 35.5 mM, 3.4 units and 40 mM, 1.1 units for guaiacol. Compared to soluble-HRP, the C3-HRP exhibited a greater oxidizing affinity toward several phenolic compounds (Guaiacol, *o*-dianisidine, *o*–phenylenediamine, pyrogallol, *p*-aminoantipyrine). In comparison with soluble-HRP, the C3-HRP showed increased stress tolerance with Triton X-100, urea, metals, isopropanol, and dimethyl sulfoxide. The C3-HRP removed methyl orange more effectively compared to soluble-HRP.

## Introduction

Rapid industrial expansion and population increase have resulted in several environmental hazards, one of them being water contamination^1^. In this instance, incorrect disposal methods for organic pollutants, such as synthetic dyes, may harm the environment and public health^2,3^. Due to the massive number of dyes used in the textile, plastic, printing, and pharmaceutical sectors, the development of innovative decolorization processes is essential^4,5^. Furthermore, this procedure is required due to the intricate chemical structures of organic dyes and their resistance to traditional methods of treating water, such as advanced oxidation, pre-concentration, and coagulation^6^. Given these circumstances, the use of enzymes has been demonstrated to be a practical dye removal technique with several advantages, including high efficiency and low treatment requirements^7,8^.

Many aromatic compounds were oxidized by peroxidases, which were linked to electron oxidation^9–11^. Usually, peroxidases transform oxidized aromatic compounds into products that aren’t very soluble in water. Peroxidases are used in the bioremediation of phenol derivatives and dye decolorization^12,13^. Azo dyes elute into wastewater generated by various industries, including the textile and paper sectors. Some reports have shown that azo dyes are typically thought to be harmful chemicals that cause cancer^14,15^. As a result, wastewater cannot be released onto agricultural land until azo dyes have been removed^16^. The oxidation of azo dyes into less dangerous compounds is normally done by HRP^17,18^.

There are certain restrictions with enzymes that may hinder their industrial utilization. The viability of using enzymes in industrial processes is dependent on some factors, encompassing enzyme activity, selectivity, and specificity towards the intended industrial substrate, enzyme purity, and enzyme stability under industrial circumstances^19–22^. Free enzymes might be difficult to extract from the reaction media because they are soluble in water. As a result, high-activity enzymes can be lost, and each reaction cycle requires the addition of a fresh enzyme load. Enzymes become less effective as catalysts as a result, possibly increasing process costs^23^. Immobilization techniques can be used to make greater use of these biocatalysts. Additionally, the immobilization of enzymes allows for continuous processing, shields the product from enzymatic contamination, and increases enzyme stability, which improves the economics of the process^24,25^. Numerous solid materials, both organic and inorganic, have been used to investigate enzyme immobilization^26,27^. An et al. reported that although organic materials lack the necessary chemical and thermal stability for usage in industry and may present a danger of enzyme toxicity, enzymes immobilized in these materials can retain their high activity^28^. Inorganic materials, such as minerals^29,30^, carbon-based materials^31^, and ceramics^32^, can be used to get around these restrictions. The stability of enzymes can be enhanced by inorganic materials since they provide better mechanical, microbiological, and thermal resistance than organic materials^33^.

Ceramic supports are among the inorganic materials that offer many benefits, including high resistance to chemicals, mechanical shocks, and heat, a large surface area, suitable pore size, and biocompatibility^34–36^. Titania^37^, Alumina^38^, zirconia^39^, silica^32,40^, and many more materials are examples of ceramic materials that are employed. Ceramic properties make it potential to make use of immobilized enzymes^33^. In this study, HRP was immobilized on ceramic materials as inorganic support. Immobilization conditions were optimized; physicochemical properties of free and immobilized enzymes were investigated. The immobilized enzyme was subsequently applied to remove the azo dye.

## Materials and methods

### Chemicals

Guaiacol, hydrogen peroxide, *O*-dianisidine, *O*-pheylenediamine, pyrogallol,* P*-aminoantipyrine, urea, Triton X-100, isopropanol, dimethylsulphoxide, and methyl orange were purchased from Sigma-Aldrich.

### Horseradish peroxidase (HRP)

Mohamed et al. purified HRP earlier^41^. (Specific activity: 7961 U/mg protein and fold purification: 8.71).

### Assessing peroxidase activity

Miranda et al.^42^ reported that the peroxidase activity was evaluated in the presence of 20 mM sodium acetate buffer, pH 6.0, 8 mM H_2_O_2_, 40 mM guaiacol, and HRP-enzyme. The change in absorbance (A_470_) 1.0/min is considered one unit.

### Immobilization process

End-over-end rotation at 90 rpm was used to immobilize the enzymes using ceramic (C) (2–10 mg), and HRP (3–24 units) at room temperature overnight. The supernatant was drawn and C-HRP was washed with distilled water and dried under lyophilization at − 50 °C for one day and stored at 4 °C for further use. Using the following formula, the immobilization efficiency of the immobilized enzyme was determined:

$$Immobilization\,\,efficiency \left(\%\right)=\frac{Activity\,of\,immobilized\,enzyme}{Initial\,activity\,of\,soluble\,enzyme}\times 100$$

### Surface structure

With an accelerating voltage of 20 kV, an energy dispersive X-ray (EDX) equipped with a Holland Field Emission Scanning Electron Microscope (FE-SEM, Quanta FEG250) was used to analyze the surface morphology of C3 and C3-HRP.

### FTIR-analysis

C3 and C3-HRP spectra were obtained using the Bruker ALPHA-FTIR-Spectrometer (400–4000 cm^1^).

### Reusability of immobilized enzyme

After a complete enzyme assay, the C3-HRP was removed from the reaction mixture and washed with buffer, and the reusability of the C3-HRP was ascertained. It was then utilized in the same reaction mixture one more time.

### pH optimum

The optimal pH of both soluble and C3-HRP was determined at various pH ranges (4.0–8.0) and the residual activity was measured under standard assay conditions.

### Temperature optimum and stability

The standard assay was used to assess the optimal temperature of both soluble-HRP and C3-HRP at various temperatures (20–70 °C). In the temperature stability assay, the C3-HRP or the soluble-HRP was incubated for one hour at various temperatures (30–70 °C) before substrate addition, followed by cooling in ice bath, the remaining activity was measured under standard assay conditions.

### Determination of Km and Vmax values

The Km and Vmax values of C3-HRP and soluble-HRP were calculated using H_2_O_2_ or guaiacol at different concentrations by Lineweaver–Burk plots.

### Effect of metals and some compounds

The effect of some metal ions (Mg^2+^, Ca^2+^, Al^3+^, Cu^2+^, Ni^2+^, Zn^2+^, Hg^2+^) and some denaturation compounds (Urea, Triton X-100, isopropanol, dimethyl- sulfoxide) on the activity of HRP and C3-HRP was measured and the residual activity was measured under standard assay conditions.

### Decolorization of methyl orange

The reaction mixture in 5.0 ml containing 0.05 M of the methyl orange, 0.05 M sodium acetate buffer pH 6.0, 0.008 M H_2_O_2_ and 20 units of HRP or C3-HRP. At one-hour intervals, 1.0 ml of the mixtures was sampled while they were shaking at 100 rpm. At 465 nm, the absorbance was measured.

$$Decolorization \left(\%\right)=\left[\frac{\left(A0-At\right)}{A0}\right]\times 100$$

The methyl orange absorbance is denoted by A0, while the treated methyl orange absorbance is represented by At.

All experimental procedures were carried out in compliance with relevant guidelines.

## Results and discussion

**Fig. 1:**
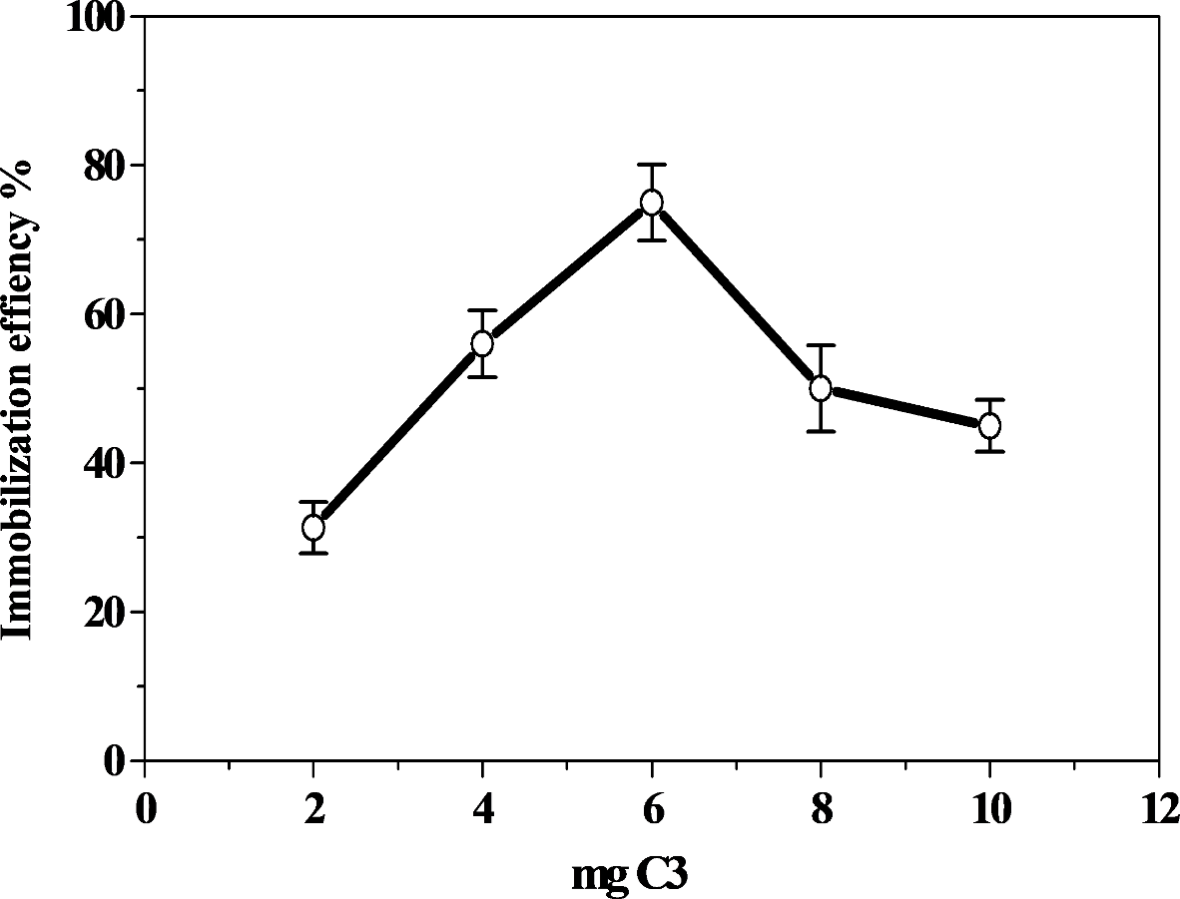
Effect of C3 concentration on the immobilization efficiency % of HRP.

**Fig. 2:**
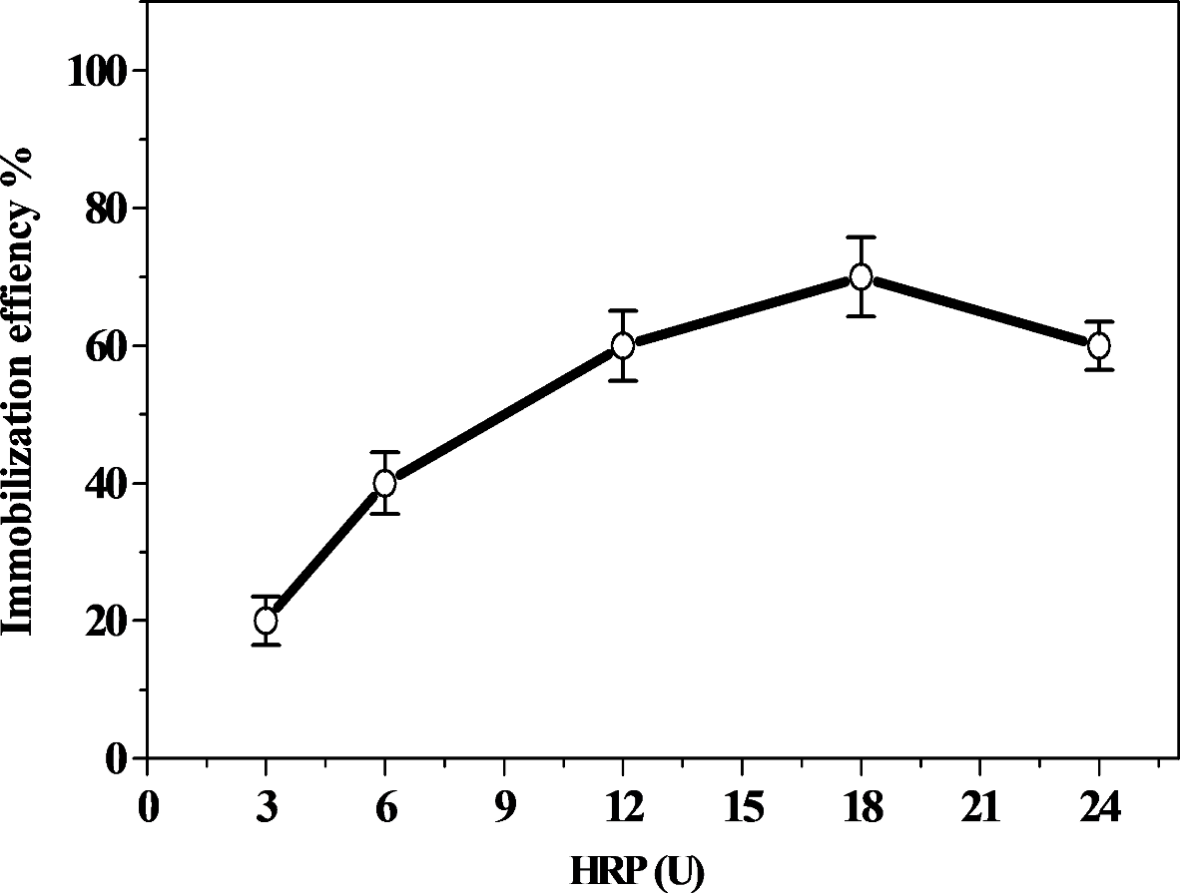
Effect of HRP concentration on the immobilization efficiency.

Five types of ceramic materials (wt% CB = bypass; GC = glass cullet = 100%) with different concentrations of oxidized metals (SiO_2_, CaO, Al_3_O_3_, Fe_2_O_3_ MgO, Na_2_O and K_2_O) were used as inorganic supports for immobilization of HRP (Table 1). The highest immobilization efficiency % was detected for C3 (Table 2). Consequently, the optimization of the immobilization conditions was performed on C3. The effect of different concentrations of C3 on the immobilization efficiency % of HRP was carried out (Fig. 1). The highest immobilization efficiency % was detected at 6 mg C3 (75%). The lowest immobilization efficiency (31%) was observed at low amount of C3 (2 mg). One possible explanation for this decrease in immobilization efficiency may be due to a low amount of enzyme adsorbed onto C3. The influence of HRP concentration on the immobilization efficiency of HRP was performed at 6 mg C3 (Fig. 2). At 18 enzyme units, the maximum immobilization efficiency percentage (71%) was found. A minor reduction in the immobilization efficiency (60%) was observed when overloading the enzyme concentration (24 units). This decrease can be the result of an excessive build-up of the enzyme on the C3, which would decrease the diffusion of the substrate^43^. Similarly, high yields (74.3–98%) were obtained by immobilizing soybean peroxidase (SBP) into various silica-ironoxide^44^. However, immobilization of enzymes typically results in a loss of activity; findings reported utilizing aminopropyl glass beads as support indicated that, following immobilization, the activity yields of horseradish peroxidase and SBP were (3%) and (35%), respectively^45,46^.

**Table 1:** Chemical composition of solid wastes in different ceramic batches (wt% CB + GC = 100%). CB = bypass; GC = glass cullet.

Batch No.	Solid waste	Composition wt%							
	CB	GC	SiO_2_	CaO	Al_2_O_3_	Fe_2_O_3_	MgO	Na_2_O	K_2_O
C1	10	90	49.66	35.99	1.79	2.59	0.80	8.68	0.49
C2	20	80	49.64	36.13	2.55	1.80	0.70	8.68	0.50
C3	30	70	49.09	36.59	2.58	1.83	0.81	8.57	0.53
C4	40	60	48.70	37.03	2.59	1.87	0.80	8.49	0.52
C5	50	50	48.31	37.93	2.57	1.95	0.82	7.91	0.51

**Table 2:** Effect of different ceramic batches on the immobilization efficiency of HRP.

Ceramic batches	immobilization efficiency %
C1	45
C2	62
C3	77
C4	65
C5	70

**Fig. 3:**
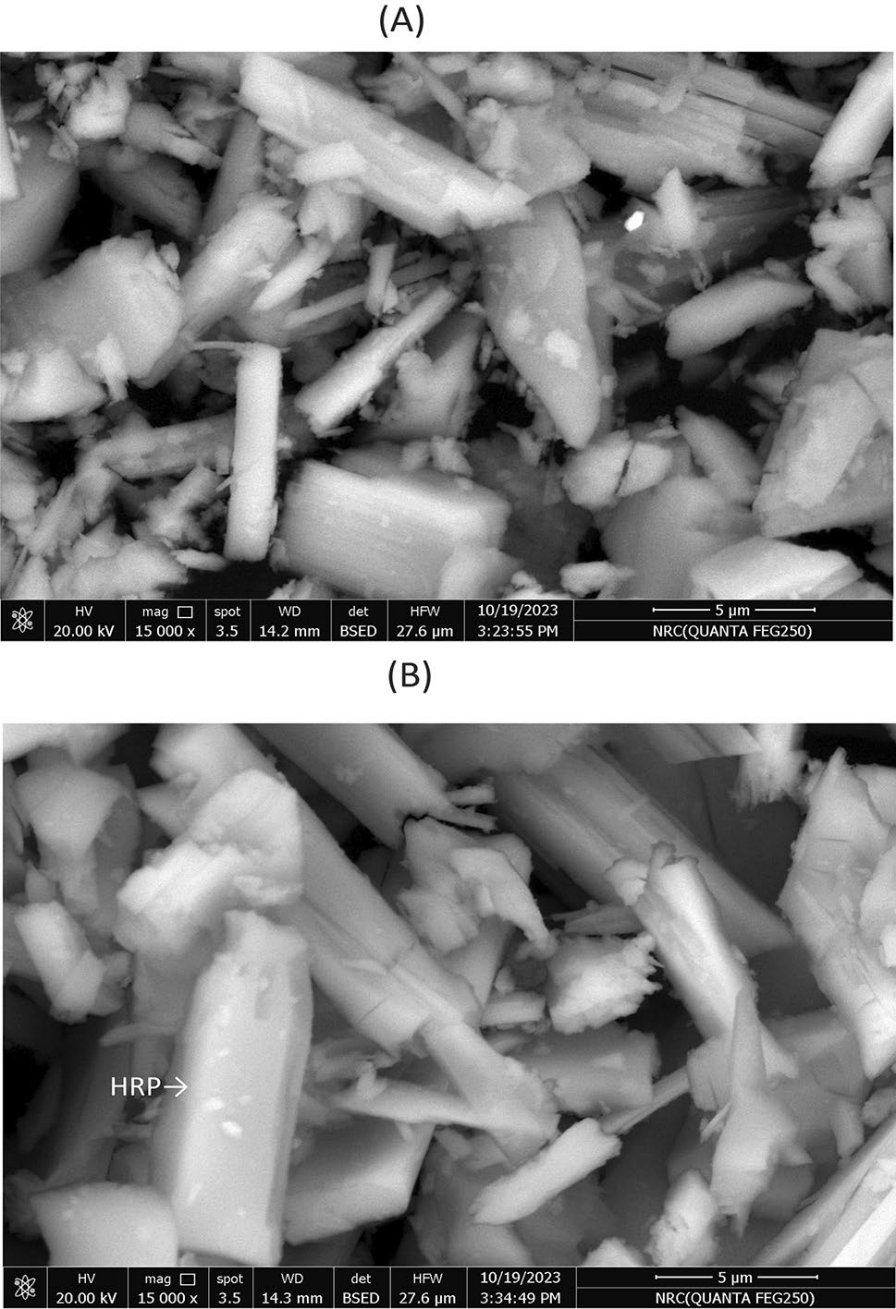
SEM microimages of the C3 (**A**) and immobilized C3-HRP (**B**).

**Fig. 4:**
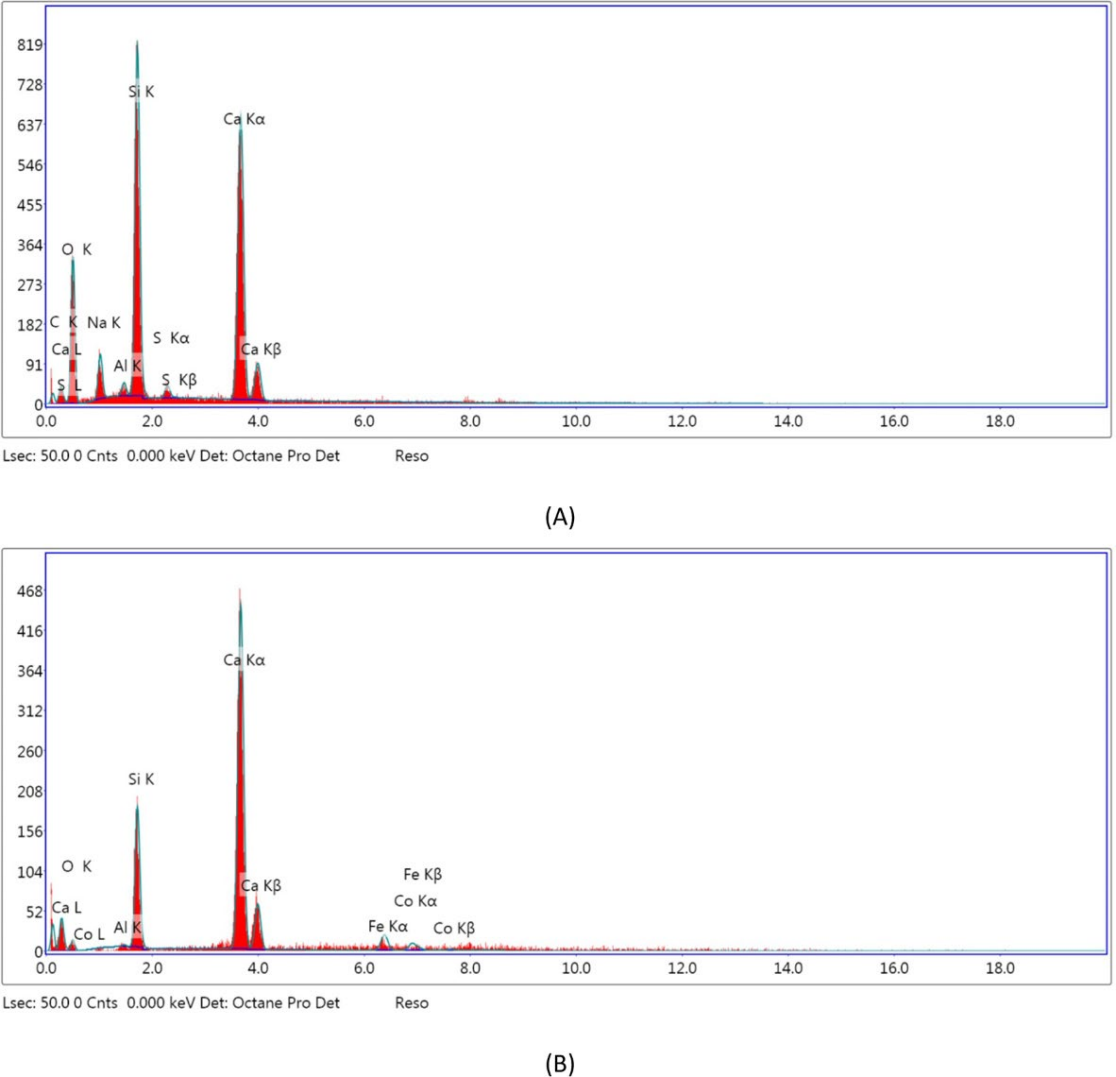
EDX analysis of C3 (**A**) and C3-HRP (**B**).

Figure 3 shows the SEM for C3 both before and after immobilization of HRP. Before enzyme immobilization, the primary characteristic of C3 was homogeneous, uniform bulk crystallization of ceramic particles in a compact structure (Fig. 3A). After enzyme immobilization, this texture changed, where the enzyme covered the pores of the C3 (Fig. 3B). This outcome demonstrates the high effectiveness of the process of enzyme adsorption. Considering the EDX study for C3, there is a rise in Si and Ca (Fig. 4A). Moreover, when the enzyme was immobilized on the surface of C3, new peaks of Fe and Co were observed (Fig. 4B). Fe may be related to peroxidase, which is present in the enzyme’s active site. Similarly, The EDX analysis of chitosan-clay composite beads did not include the Cu element. After immobilization of laccase, since Cu is a common element found in laccase active sites, the presence of Cu verified the existence of laccase^47^.

**Fig. 5:**
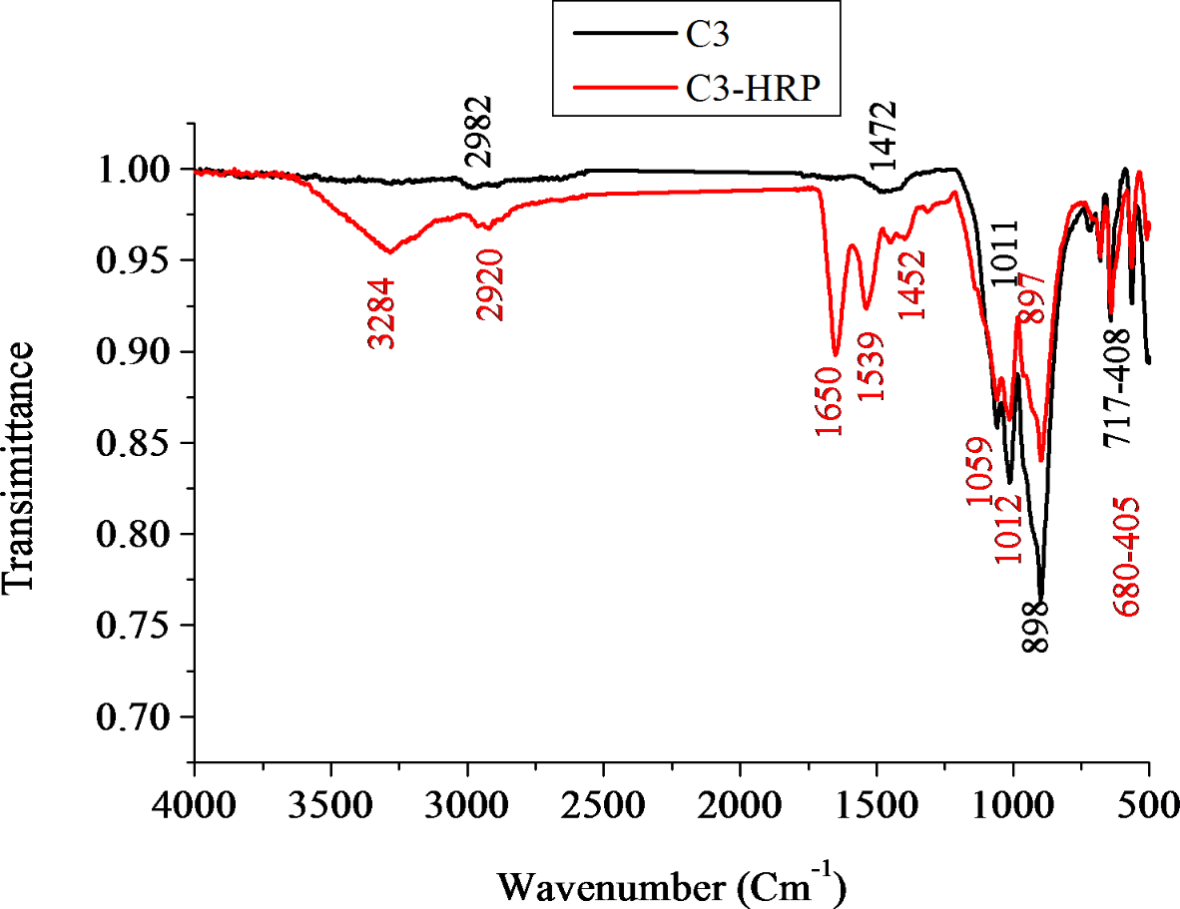
FTIR spectra of the C3 and C3-HRP.

FTIR spectra of native C3 and C3-HRP are presented in Fig. 5. For C3, the bands at 3284, and 897 cm^−1^ are attributed by Si–OH on the diatom bio-silica surface. In immobilized HRP onto NH2-modified magnetic Fe_3_O_4_/SiO_2_ particles, the typical absorption of Fe–O vibration is detected at 558 cm^−1^ as present in FTIR of C3^48^. Al-O peaks were detected at 820–900 cm^−1^ and 705–780 cm^−1^ in alkaline cement as detected by García-Lodeiro^49^. These peaks were also detected in C3. As with HRP, the amide groups are absorbed/appear at 1650, 1452, and 1059 cm^−1^, respectively. These groups include amide I (C=O stretching), amide II (N–H deformation), and amide III (C–N stretching and N–H bending). These groups’ existence suggests an amidation response and validates HRP’s immobilization on the C3 support, which was discovered by Chang and Tang^48^, Mohamed et al.^50^. Further after immobilization process, the bands/peaks at 2928, 1472, 1011, 898, and 717–408 cm^−1^ are shifted to 2920, 1452, 1012, 897, and 680–405 cm^−1^, respectively. Overall, the C3-HRP bands and structure’s shifting, overlapping, intensity fluctuations, and peroxidase functional groups point to a possible interaction between the immobilized HRP and the C3 support. These results were previously reported^51^.

**Fig. 6:**
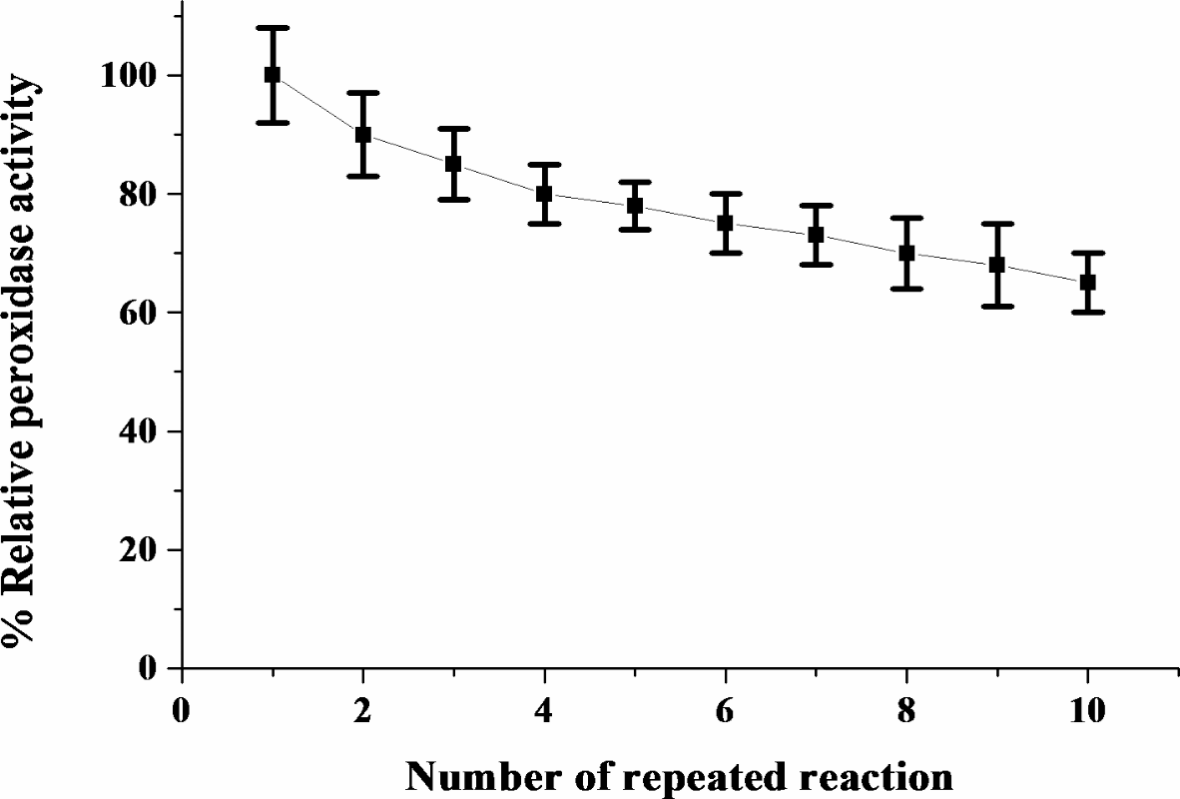
Reuses of the C3-HRP.

Reusable enzyme is one of the greatest cost-effective benefits of immobilized enzymes versus free enzymes. As shown in Fig. 6, the C3-HRP’s reusability was assessed throughout ten reuses in the current study. After ten reuses, the C3-HRP maintained (64%) of its initial activity, according to the results, indicating that it was highly stable and could be reused multiple times. The reduction in the enzyme activity over multiple reuses is may due to the accumulation of the products obstructing substrate access to the active site^45,52,53^. Similarly, immobilized SBP was reused for four cycles without significantly losing its initial activity and retained (42%) of its initial activity after ten cycles^44^. With SBP immobilized on corncob powder, César et al.^54^ saw (40%) reduction in activity after just four cycles, whereas Prokopijevic et al.^55^ observed (78%) loss during the sixth cycle using glycidyl methacrylates. HRP was rendered more reusable by immobilizing it on graphene oxide-SiO2, as demonstrated by the retention of (70%) of initial activity after ten cycles^56^.

**Fig. 7:**
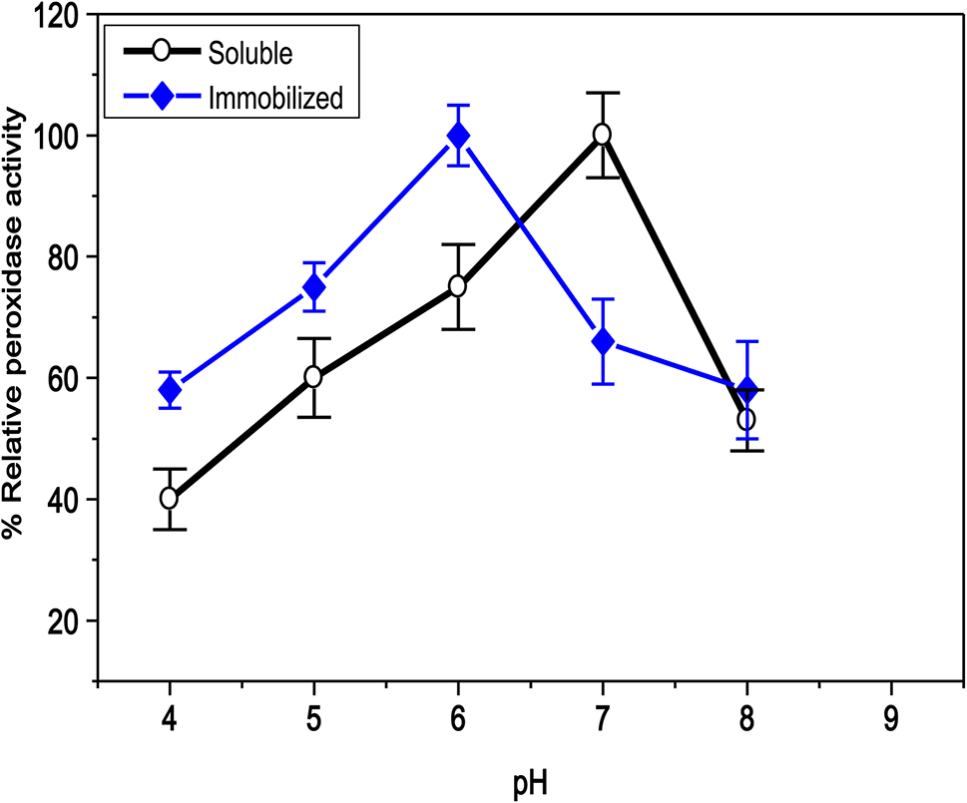
pH optima for soluble HRP and C3-HRP.

**Fig. 8:**
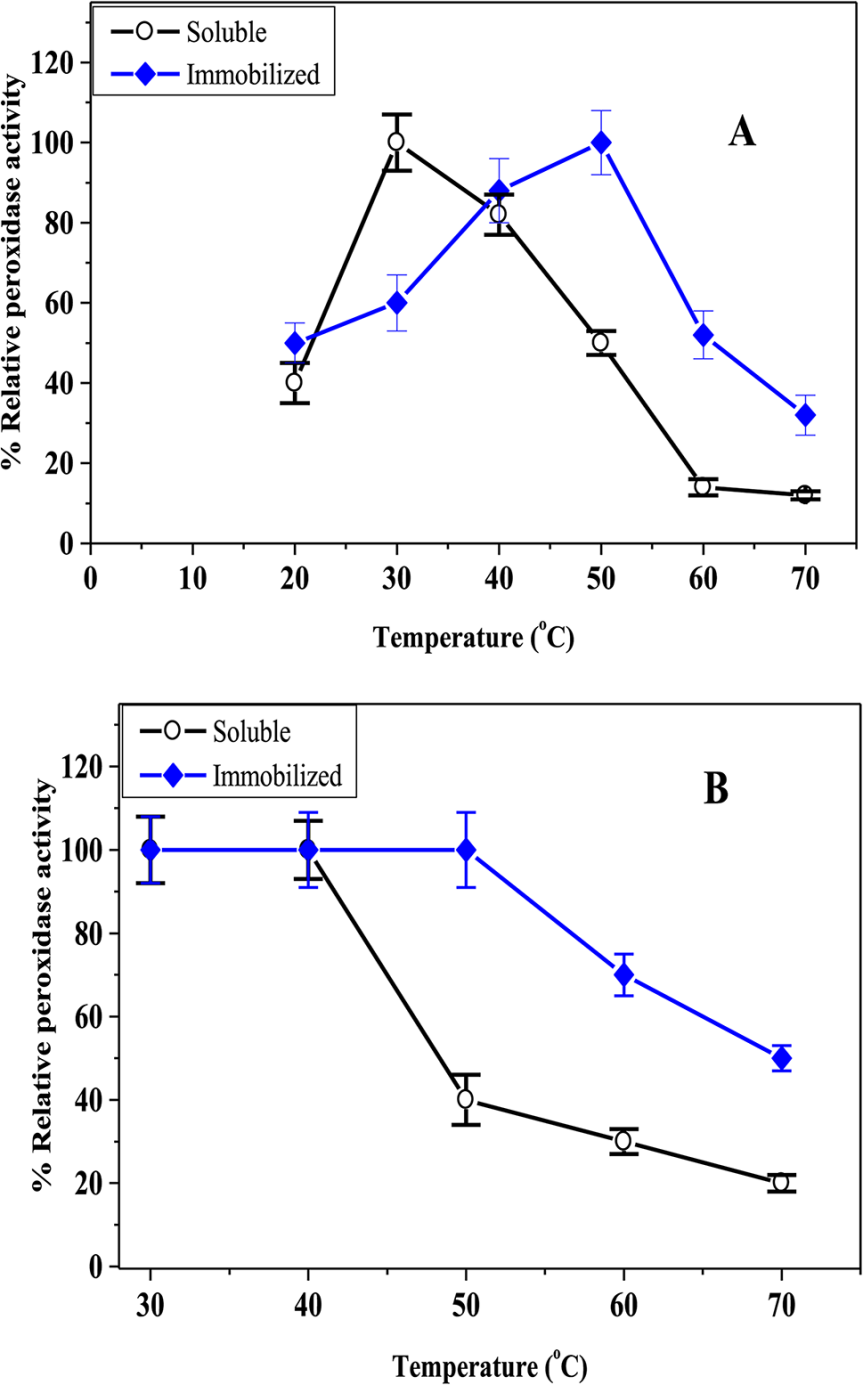
Temperature-optimum (**A**) and thermal-stability (**B**) of the soluble HRP and C3-HRP.

As illustrated in Figs. 7 and 8A and B, the immobilized C3-HRP was found to have the optimal pH, temperature, and thermal stability at pH 6.0, 50 °C, and (30–50 °C), while the soluble-HRP was found to be at pH 7.0, 30 °C, and (30–40 °C), respectively. These findings suggest that C3-HRP may be more thermally stable than free-enzyme and be able to withstand the effects of the acidic medium. The optimum pH of the immobilized HRP onto a functionalized reduced graphene oxide-SiO2 and free HRP was at pH 7.0. Compared to the free HRP, the immobilized HRP was more stable against pH changes^56^. Furthermore, at a pH of 7.0, the oil removal efficacy value peaked at (76.4%) for horseradish peroxidase immobilized on the silane-modified ceramics^57^. According to Donadelli et al.^44^, the optimal temperature for SBP on silica-coated superparamagnetic iron oxide nanoparticles is 60 °C, which is higher than the temperature for the free enzyme. Ninety % of the initial activity was retained by the immobilized HRP onto a functionalized reduced graphene oxide-SiO_2_ after 90 min at 40 °C, but only (70%) of the initial activity persisted for the free HRP^56^. These findings suggested that the immobilization may restrict the unfolding and non-specific aggregation of enzymes. On the contrary, the activity of free and HRP immobilization on the silane-modified ceramics was not influenced by temperature during the oil removal^57^.

**Fig. 9:**
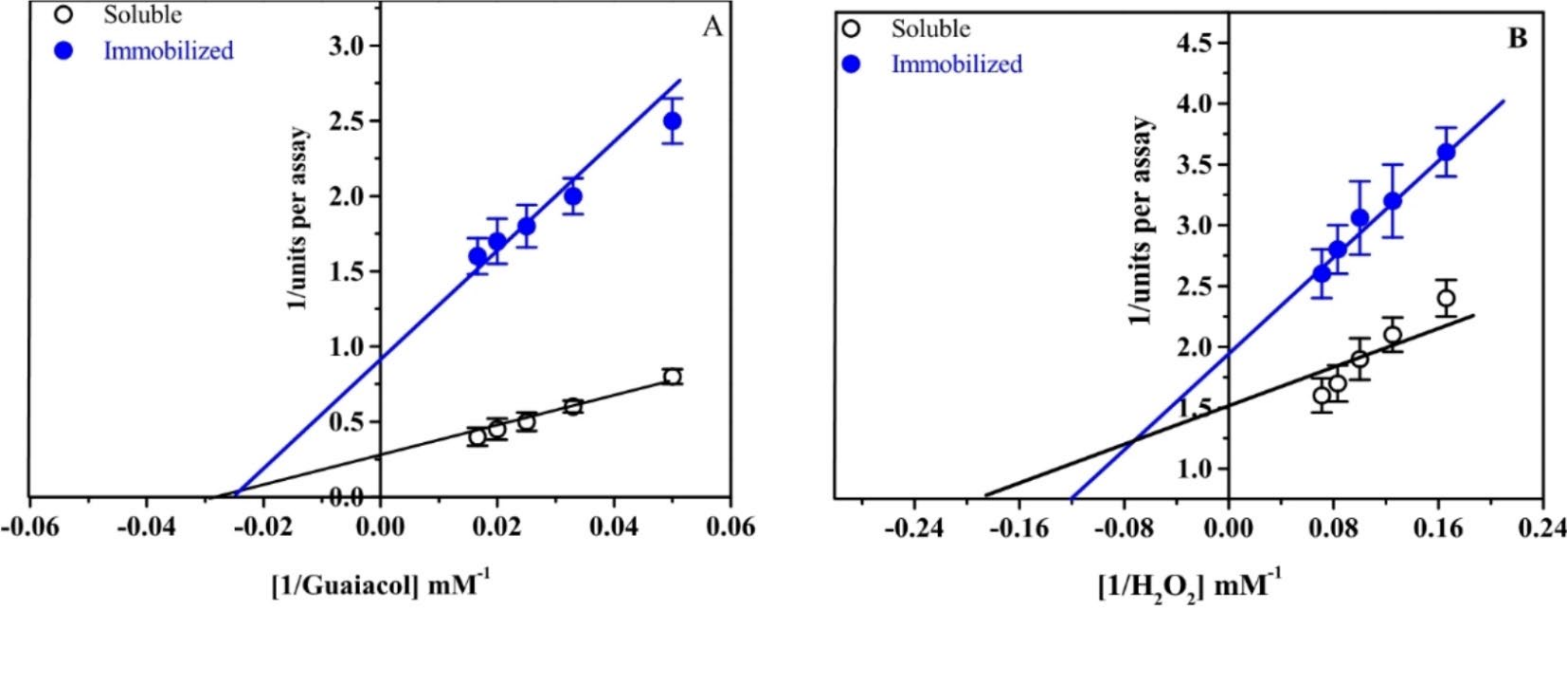
Lineweaver–Burk plots of soluble HRP and C3-HRP reaction velocities to guaiacol (**A**) and H_2_O_2_ (**B**) concentrations.

Additionally, the Km and Vmax of soluble HRP and C3-HRP were, respectively, 5.5 mM, 0.66 units and 8 mM, 0.52 units for hydrogen peroxide (H_2_O_2_) and 35.5 mM, 3.4 units and 40 mM, 1.1 units for guaiacol (Fig. 9A and B), indicating a decreased affinity of the immobilized enzyme for substrates. Similarly, the apparent Km value of the immobilized HRP onto NH2-Modified Magnetic Fe_3_O_4_/SiO_2_ is slightly greater than that of free HRP^48^. On the contrary, the Km value of the immobilized HRP onto a functionalized reduced graphene oxide-SiO_2_ was lower than that of the free HRP using 4-aminoantipyrine as substrate^56^. These modifications in the kinetic properties of immobilized enzymes may be attributed to changes in substrate diffusion and the catalytic site^58,59^. Table 3 shows that C3-HRP had a greater oxidizing affinity than soluble-HRP for some phenolic substrates (Guaiacol, *o*-dianisidine, *o*–phenylenediamine, pyrogallol, *p*-aminoantipyrine) that were evaluated. This suggests that the enzyme’s substrate-binding site structure was altered or improved.

**Table 3:** Specificity of the soluble HRP and C3-HRP for substrates. Values with different superscripts (a, b) were significant different.

Substrate	Relative activity %	
	Soluble-HRP	C3-HRP
Guaiacol	100 ± 4.1^a^	100 ± 8.0^a^
*o*-Dianisidine	68 ± 6.4^b^	80 ± 19.2^b^
*o* -phenylenediamine	80 ± 5.0^b^	126 ± 10.2^b^
Pyrogallol	26 ± 2.8^b^	66 ± 11.7^b^
*p*-Aminoantipyrine	33 ± 3.8^b^	102 ± 8.2^b^

Table 4 displays the impact of metal ions on the activity of enzymes in soluble HRP and C3-HRP. Comparing the examined metals to soluble HRP, none of them had much of an inhibitory effect on C3-HRP. On C3-HRP, Mg^2+^, Al^2+^, Cu^2+^, and Ni^2+^ had no discernible effects. Furthermore, the soluble enzyme is more strongly inhibited by Hg^2+^. According to these findings, the immobilization procedure strengthened the enzyme’s structure to withstand the action of metals, and C3-HRP was utilized to treat wastewater that has significant concentrations of heavy metals. The effects of metal ions on horseradish peroxidase immobilized on the silane-modified ceramics revealed that Fe^2+^, Zn^2+^, and Mg^2+^ enhanced the effectiveness of oil removal, whereas Cu^2+^ and Mn^2+^ reduced it. The effectiveness of oil removal was not affected by Ca^2+^^57^.

**Table 4:** Effects of several metal ions on the soluble HRP and C3-HRP at a concentration of 10 mM. Values with different superscripts (a, b) were significant different.

Metals	Relative peroxidase activity %	
	Soluble-HRP	C3-HRP
None	100 ± 4.3	100 ± 3.1^a^
Mg^2+^	72 ± 18.5^b^	100 ± 6.0^b^
Ca^2+^	80 ± 20.2^b^	87 ± 5.1^b^
Al^3+^	75 ± 11.5^b^	100 ± 5.0^b^
Cu^2+^	51 ± 10.5^b^	102 ± 5.6^a^
Ni^2+^	69 ± 5.2^a^	105 ± 3.3^b^
Zn^2+^	65 ± 6.2^a^	80 ± 4.3^b^
Hg^2+^	30 ± 5.8^a^	55 ± 3.1^b^

Table 5 shows how certain chemicals affect the activity of soluble and immobilized HRP. The soluble-HRP retained (20%) and (9%) of its initial activity at 2.0 and 4.0 M urea, whereas the immobilized C3-HRP retained (40%) and (25%). This shows that the HRP structure was altered throughout the immobilization process to withstand the effect of urea, which causes protein unfolding and reduces enzyme activity. Similarly, HRP–nonwoven polyester fabric coated with chitosan showed greater resistance against urea than the soluble HRP^60^. The C3-HRP’s activity dropped to (33%) at (10%) Triton X-100, whilst the soluble-HRP only held onto (12%) of its activity. An extremely significant finding is that the immobilized C3-HRP is resistant to Triton X-100, indicating that it may withstand the detergent action encountered during wastewater treatment. Compared to the soluble HRP, the wool-HRP exhibited a significantly higher degree of stability when exposed to Triton X-100^61^. During the wastewater treatment process, the peroxidase activity is adversely affected by organic solvents. Under (10%) isopropanol and dimethyl sulfoxide treatment, the C3-HRP kept (62%) and (83%) of its activity and the soluble-HRP retained (45%) and (25%). These results indicate that the synthesized C3-HRP is capable of effectively withstanding the organic solvents already present in wastewater. Mohamed et al.^60^ demonstrated that chitosan-coated HRP-nonwoven polyester fabric exhibited more residual activity in organic solvents than did the free enzyme.

**Table 5:** Effect of some compounds on the soluble HRP and C3-HRP. Values with different superscripts (a, b) were significant different.

Substrate	Concentration	Relative activity %	
		Soluble-HRP	C3-HRP
None	–	100 ± 5.3^a^	100 ± 4.2^a^
Urea	2 M	20 ± 1.8^b^	40 ± 2.2^b^
	4 M	9 ± 0.8^b^	25 ± 1.2^b^
Triton X-100	10%	12 ± 1.0^b^	33 ± 1.2^b^
Isopropanol	10%	45 ± 1.7^b^	62 ± 2.7^b^
Dimethylsulphoxide	10%	25 ± 1.2^b^	83 ± 3.7^b^

Urea, Triton X-100, isopropanol and dimethylsulphxide are used as protein unfolding compounds, detergent, and solvents and their effect on enzyme activity, respectively.

Methyl orange, an azo dye, was decolored using HRP soluble and immobilized forms (Table 6). For removing methyl orange, the immobilized form outperformed the soluble version. Following a 5-h incubation period, (45%) of the dye was eliminated in soluble form, while (82%) of the methyl orange was decolorized in immobilized form. However, the methyl orange was eliminated by (66%) and (60%), respectively, by the soluble peroxidases from horseradish and Ficus sycomorus latex^12^. Good efficiency in malachite green removal was achieved by immobilizing SBP onto silica-coated superparamagnetic iron oxide nanoparticles^44^. Also immobilized HRP on RGO-SiO_2_ nanocomposite had high potential toward different dyes compared to free enzyme^56^.

**Table 6:** Impact of soluble HRP and C3-HRP on methyl orange elimination. Values with different superscripts (a, b) were significant different.

Time (h)	% removal of dye	
	Soluble HRP	C3-HRP
1	18 ± 0.28^b^	33 ± 0.12^b^
2	25 ± 0.24^b^	47 ± 0.42^b^
3	36 ± 0.44^b^	65 ± 0.62^b^
4	40 ± 0.54^b^	73 ± 0.65^b^
5	45 ± 0.52^b^	82 ± 0.72^b^

## Conclusion

Due to the good mechanical and electrostatic interaction properties of the ceramic, the immobilized C3-HRP was well recyclable and maintained their catalytic activity up to 10 runs which is an important factor for industrial processing. Changes in the C3-HRP are linked to enhanced heat resistance of enzymes. The technique of immobilization enhanced the catalytic efficiency, enhanced the hydrolysis reaction, and increased the affinity for phenolic substrates. According to the findings, immobilizing HRP on C3 was a great method for creating an active biocatalyst that would remove the azo dye.

## Data Availability

The datasets generated during and/or analyzed during the current study are available from the corresponding author on reasonable request.
